# Beyond Commuting: Ignoring Individuals’ Activity-Travel Patterns May Lead to Inaccurate Assessments of Their Exposure to Traffic Congestion

**DOI:** 10.3390/ijerph16010089

**Published:** 2018-12-30

**Authors:** Junghwan Kim, Mei-Po Kwan

**Affiliations:** Department of Geography and Geographic Information Science, Natural History Building, 1301 W Green Street, University of Illinois at Urbana-Champaign, Urbana, IL 61801, USA; jk11@illinois.edu

**Keywords:** traffic congestion, activity-travel patterns, real-time traffic data, the uncertain geographic context problem (UGCoP), the neighborhood effect averaging problem (NEAP)

## Abstract

This research examines whether individual exposures to traffic congestion are significantly different between assessments obtained with and without considering individuals’ activity-travel patterns in addition to commuting trips. We used crowdsourced real-time traffic congestion data and the activity-travel data of 250 individuals in Los Angeles to compare these two assessments of individual exposures to traffic congestion. The results revealed that individual exposures to traffic congestion are significantly underestimated when their activity-travel patterns are ignored, which has been postulated as a manifestation of the uncertain geographic context problem (UGCoP). The results also highlighted that the probability distribution function of exposures is heavily skewed but tends to converge to its average when individuals’ activity-travel patterns are considered when compared to one obtained when those patterns are not considered, which indicates the existence of the neighborhood effect averaging problem (NEAP). Lastly, space-time visualizations of individual exposures illustrated that people’s exposures to traffic congestion vary significantly even if they live at the same residential location due to their idiosyncratic activity-travel patterns. The results corroborate the claims in previous studies that using data aggregated over areas (e.g., census tracts) or focusing only on commuting trips (and thus ignoring individuals’ activity-travel patterns) may lead to erroneous assessments of individual exposures to traffic congestion or other environmental influences.

## 1. Introduction

Traffic congestion has long been a serious transportation-related issue that people confront in their daily life in the U.S. [1,2,3]. Drivers in the U.S. wasted 7 billion hours on roads in 2015 due to delays caused by traffic congestion [4]. Moreover, it is expected that traffic congestion will intensify in the future as more people will move to urban areas [5]. Transportation and health researchers have thus considered traffic congestion exposure as a critical factor that influences individuals’ physical and mental health. For example, a number of studies revealed that higher exposure to traffic congestion may be associated with escalated heart rate and blood pressure [6,7], heightened urinary catecholamine (a stress-related hormone) [8], and negative health outcomes [9,10,11]. In addition to these physical tolls, studies have also shown that exposures to traffic congestion may be linked to psychological stress [12,13,14,15,16,17] and reduced well-being [18,19]. Furthermore, some studies have argued that longer commuting time, which is worsened by traffic congestion, may harm people’s work-family balance [20] or even increase the likelihood of being involved in domestic violence [21].

To accurately assess the effects of traffic congestion exposures on human health, it is important to accurately measure individual exposures to traffic congestion. Specifically, in terms of measuring traffic congestion exposures, most previous studies used area-based aggregate data (e.g., [9,21,22]) or focused only on commuting trips (and thus disregarded non-commuting trips) (e.g., [11,18,19]). We argue that these approaches in previous research may lead to erroneous assessments of individual exposures to traffic congestion, which may in turn lead to erroneous evaluations of the health impacts of traffic congestion because they did not consider individuals’ unique activity-travel patterns.

To address the limitations of previous studies, this research examines whether individual exposures to traffic congestion are significantly different between assessments obtained with and without considering individuals’ activity-travel patterns in addition to commuting trips. We used crowdsourced real-time traffic congestion data and the activity-travel data of 250 individuals in Los Angeles to compare these two assessments of individual exposures to traffic congestion. The results indicate that ignoring individuals’ activity-travel patterns may lead to inaccurate assessments of their exposures to traffic congestion.

## 2. Limitations of Previous Studies

Previous studies on traffic congestion have several limitations, some of which this study seeks to address. First, existing research used data aggregated over areas such as metropolitan areas or zip (postal) code areas. For example, Levy et al. [9] assessed the health impact of PM_2.5_ exposure associated with traffic congestion on mortality and monetized the value of mortality risk in 83 metropolitan areas in the U.S. By using zip code areas in the Los Angeles Metropolitan Area, Beland and Brent [21] noted that traffic congestion may lead to a higher risk of being involved in domestic violence. Brauer et al. [22] examined people’s traffic-related air pollution “exposure zones” (defined as a 500-m buffer zone from highways or a 100-m buffer zone from major arterials) and observed that 32% of people in Canada live in exposure zones.

Although these studies provide useful insights into the health impacts of traffic congestion, their estimations of traffic congestion exposure may be erroneous. Part of the reason for the error is because the units of analysis are areal units for which individual data are aggregated (e.g., buffer zones, zip code areas, or metropolitan areas); and the modifiable areal unit problem (MAUP) may contribute to some of such error. This means that previous studies presumed that individuals in the same areal unit are exposed to the same level of traffic congestion when estimating traffic congestion exposure. However, this assumption is problematic because each individual may have distinctive activity-travel patterns and thus may have different levels of exposure to traffic congestion and experience different health impacts [23,24]. In other words, since individuals have idiosyncratic activity-travel patterns, different individuals may be exposed to different levels of traffic congestion in complex and unique ways even when they live in the same area.

Second, previous research focused only on commuting trips while ignoring other types of trips, where individuals may also be exposed to significant traffic congestion. For instance, Olsson et al. [25] found that commuting satisfaction may affect overall happiness based on a survey of 713 commuters. Using a 23-year longitudinal dataset of 2736 commuters, Sandow et al. [11] showed that there may be gender differences in mortality risks due to longer commuting. Despite the meaningful results of these previous studies, focusing exclusively on commuting trips while not considering other components of individuals’ activity-travel patterns may lead to erroneous estimations of their exposure to traffic congestion for the following two reasons.

First, commuting trips account for only a small proportion of individuals’ total travel. Although commuting trips seem to constitute the most significant portion of our trips at first glance, almost 70% of trips in the U.S. consists of non-work trips according to the 2009 National Household Travel Survey [26]. Thus, considering only commuting trips may result in erroneous estimations of traffic congestion exposure because people may also experience traffic congestion when undertaking other types of trips, which also comprise their travel and are overlooked by previous studies.

Second, and more specifically, individuals also experience traffic congestion when undertaking trips during non-peak hours. At first sight, it sounds reasonable that commuting trips in peak hours (e.g., 7–9 A.M., 3–6 P.M.) are the only time when individuals are exposed to traffic congestion. However, this may not be true for large cities, where serious traffic congestion occurs almost all day long in certain road segments (e.g., [27,28,29]). This suggests that individuals are exposed to traffic congestion in complex ways in their daily life. They may be exposed to different levels of traffic congestion when undertaking not only commuting trips during peak hours but also other types of travels during non-peak hours.

We thus argue that, for these two reasons, the approaches used in previous studies on individual exposure to traffic congestion and its health impact may exacerbate the uncertain geographic context problem (UGCoP). The UGCoP is a critical methodological issue, and one of the ways in which it may be encountered is when people’s exposure to environmental contexts or risk factors (e.g., traffic congestion and air pollution) is inaccurately estimated as a result of ignoring their activity-travel patterns [23,24]. Recent studies have shown that using inaccurately estimated exposures to environmental pollutants may lead to serious inferential errors or misleading results when examining the health impacts of such exposures [30,31,32,33,34,35].

Further, these approaches in previous studies may aggravate the neighborhood effect averaging problem (NEAP) that arises when individuals’ unique activity-travel patterns are ignored [36]. The NEAP suggests that when the probability distribution of residence-based exposures approximates a bell-shaped distribution, individual exposures tend to converge toward the average if individuals’ activity-travel patterns are considered when compared to exposures obtained when such patterns are not considered. As one of the manifestations of the UGCoP, the NEAP thus suggests that ignoring individuals’ activity-travel patterns may lead to erroneous exposure estimations because of neighborhood effect averaging [36]. Eventually, this may also result in inferential errors or misleading results when researchers investigate the health effects of these exposures [23,24].

In traffic congestion exposure and health studies, these two methodological issues—the UGCoP and the NEAP—arise because of the following two reasons. First, the issues are caused by the spatiotemporal variations in traffic congestion intensities. Although at first glance, the levels of traffic congestion seem to be homogeneous over space and time in large metropolitan areas, this may not be true. For example, intensities of traffic congestion in non-peak hours may not be as severe as those in peak hours (i.e., temporal variations). Also, even for the same time of day, people may be exposed to different traffic congestion intensities based on where trips occur (i.e., spatial variations). Second, the issues arise because individuals are undertaking various types of trips rather than undertaking commuting trips only. Therefore, individuals may be exposed to traffic congestion in complex spatiotemporal ways when they are undertaking commuting trips and other trips [23,37,38].

To sum up, the approaches in previous research examining the effects of traffic congestion exposure on human health largely ignore the UGCoP and the NEAP. This is because both the spatiotemporal variations in traffic congestion and individuals’ unique activity-travel patterns increase contextual complexities when measuring individual exposures to traffic congestion. These complexities may lead to erroneous assessments of individual exposures to traffic congestion if their activity-travel patterns are overlooked. Eventually, using inaccurately estimated traffic congestion exposure may exacerbate inferential errors or lead to misleading results when investigating the health effects of traffic congestion exposure.

In light of the relative ignorance of critical methodological issues like the UGCoP and the NEAP in past research on traffic congestion exposure and health, this research seeks to fill this gap. Specifically, this research examines whether individual exposure to traffic congestion is significantly underestimated when individuals’ travels beside commuting trips are ignored. To achieve this research goal, we seek to answer the following three questions in this paper by utilizing crowdsourced real-time traffic congestion data and activity-travel data of 250 individuals in Los Angeles, California.

The first question is: Do spatiotemporal variations exist in traffic congestion intensities? In other words, we will investigate whether traffic congestion intensities are different over space and time. Spatiotemporal variations in traffic congestion intensities indicate that two approaches adopted by previous studies (i.e., using data aggregated over areas like census tracts and focusing on commuting trips only) may exacerbate the UGCoP and the NEAP, which is problematic.

The second question is: Will exposures to traffic congestion based on commute-only assessments be significantly lower than those obtained from assessments that also consider individuals’ activity-travel patterns in addition to commuting trips? In other words, we will compare individuals’ exposure to traffic congestion obtained from two assessments: one that only considers commuting trips and one that also considers individuals’ activity-travel patterns in addition to considering commuting trips. If we find that exposures to traffic congestion based on commute-only assessments are significantly lower than those obtained from assessments that also consider individuals’ activity-travel patterns in addition to commuting trips, more attention is needed to address the UGCoP and the NEAP in future research on traffic congestion exposure and health.

Lastly, the third question is: How are individuals uniquely exposed to traffic congestion as a result of their activity-travel patterns? In other words, can we observe individuals’ idiosyncratic activity-travel patterns and their associated exposures to traffic congestion? Answering this question will help researchers develop an in-depth understanding of the unique characteristics of individuals’ activity-travel patterns and how such unique characteristics may affect the accurate assessment of traffic congestion exposure.

## 3. Study Area and Data

### 3.1. Study Area

**Figure 1:**
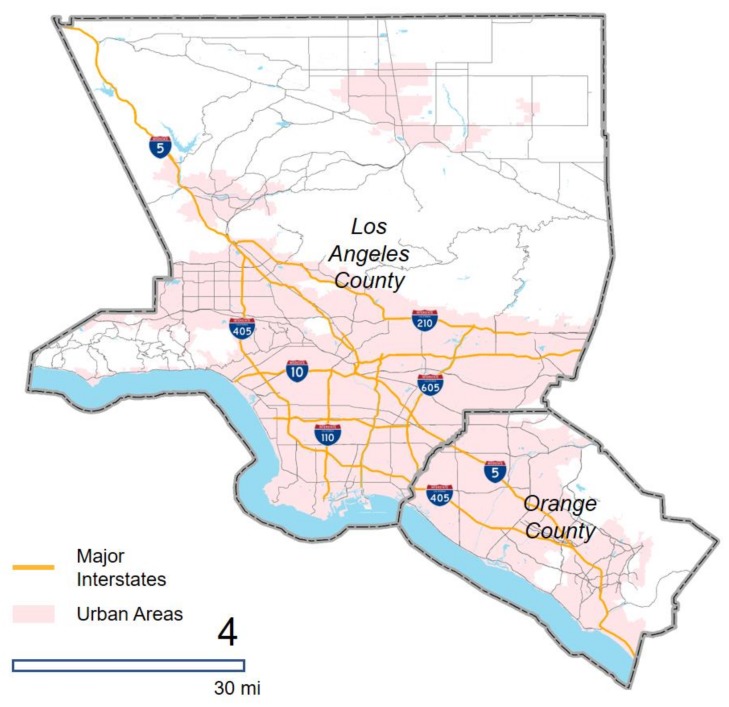
The Los Angeles Metropolitan Area and its major highways.

The study area for this research is the Los Angeles-Long Beach-Anaheim Metropolitan area in California, consisting of Los Angeles County and Orange County (Figure 1). We chose the Los Angeles Metropolitan Area for its renowned severe traffic congestion. According to a recent traffic congestion assessment study, the Los Angeles region is ranked as the first out of 297 cities in the U.S. as well as the first out of 1360 cities in the world regarding its severity of traffic congestion [29]. Additionally, the entire metropolitan area is selected as a study unit as it comprehensively captures individuals’ socioeconomic characteristics and activity-travel patterns [39].

### 3.2. Data

#### 3.2.1. Individual Activity-Travel Survey Data

Since the study seeks to obtain results based on people’s actual activity-travel behaviors (not to compare the congested and non-congested travel speeds/times of all road segments in the study area), we employed individual activity-travel survey data accessed via the Transportation Secure Data Center [40]. This individual activity-travel survey is a part of the “National Household Travel Survey California Add-On” survey conducted in 2017. The survey data were collected from around 55,800 individuals (about 26,000 households) in California. Participants were asked to report their activity-travel patterns (e.g., the location of activities, duration of activities, trip purposes, modes of travel, and the number of accompanying passengers) for one survey day and their socioeconomic attributes. Note that the survey did not collect or provide any global positioning system (GPS) data for constructing the space-time trajectories of participants’ trips. Therefore, to estimate the travel time of participants’ trips and their exposures to traffic congestion, we assumed that they used the shortest path (in terms of travel time) to travel between all locations and used the Google Maps Application Programming Interface (API) to derive the travel time for each trip based on the time of day and the shortest travel route for the trip.

We selected individuals according to the research goal as follows. First, we selected individuals whose trips were all in the study area (i.e., the Los Angeles Metropolitan Area) on weekdays. We only focused on weekdays because weekend activity-travel patterns typically consist of non-routine patterns such as recreational trips and often do not involve any commuting trips [26]. Second, we selected individuals who were actively employed because unemployed people do not undertake any commuting trips. Third, we selected individuals who made trips by driving alone without any accompanying passengers. In other words, all trips in this study were traveled by driving, and trips made by public transit and non-motorized modes (including buses, taxis, bicycles, and walking) were not considered. We focused on these individuals to control other possible travel-mode related factors that may also influence how traffic congestion exposure affects health. For instance, previous studies found that the effects of traffic congestion exposure on health may be different when individuals are drivers rather than passengers (e.g., [41,42]). Moreover, they found that the existence of accompanied passengers may affect drivers’ stress (e.g., [7,42,43]). 

Lastly, individuals who did not undertake any commuting trips or who had only commuting trips were excluded because we seek to generate two exposure assessments, one that considers only commuting trips and the other considers both commuting and non-commuting trips. Note that we define commuting trips as trips that are anchored at a workplace so that we can consider trip-chaining travel behaviors. Before this exclusion criterion was applied, there were 729 individuals in our subsample. As a result of applying this exclusion criterion, 77 individuals (11%) were excluded because they did not make any commuting trips (e.g., having a day-off from the work), and an additional 402 individuals were removed since they only made commuting trips (i.e., no other type of trips). Note that a considerable portion of the survey participants (34%, 250 individuals) reported that they made commuting as well as non-commuting trips. This provides a compelling rationale that individuals’ activity-travel patterns still should be considered to accurately assess their exposure to traffic congestion.

After this selection process, 250 individuals were finally included in the subsample used in this study. To avoid sample selections that do not have similar sociodemographic characteristics as the larger population in the Los Angeles Metropolitan Area, we compared their socio-economic attributes with those of the larger population in the study area. Note that since our research focuses on employed individuals, the statistics reported in Table 1 represent only employed workers. Overall, descriptive statistics of the selected participants showed similarity to those of the larger population in the study area. The only discrepancy we found is that the median age of the selected participants (45.2 years old) is higher than that of the larger population in the Los Angeles Metropolitan Area (39.9 years old). This can be explained by underrepresentation of the younger generations in our subsample as we focus on workers who drive their own cars. Recent travel behavior studies revealed that the younger generation (e.g., millennials) may drive less or not own cars (e.g., [44,45]). Therefore, it is likely that the younger generation may be underrepresented in our subsample.

#### 3.2.2. Real-Time Traffic Congestion Data

**Figure 2:**
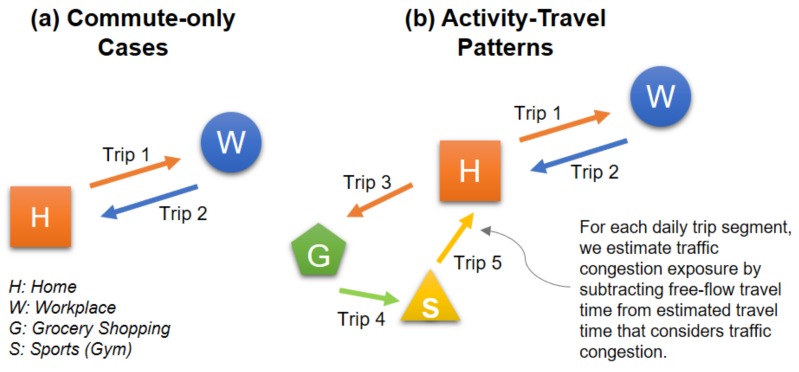
An example of estimating an individual’s exposure to traffic congestion for two types of assessments: (**a**) commute-only versus (**b**) activity-travel patterns in addition to commuting trips.

In this study, we estimate individual exposure to traffic congestion for each trip by subtracting its free-flow travel time from its estimated travel time that considers traffic congestion, following the framework used in previous studies (e.g., [29,46,47]). For example, imagine an individual who undertakes 5 trips in his or her daily life (Figure 2). This person travels from the home location to a workplace (Trip 1), goes back from the workplace to home (Trip 2), goes grocery shopping from home (Trip 3), goes to the gym after the grocery shopping (Trip 4), and finally goes back home from the gym (Trip 5). For each of these trips, by subtracting the free-flow travel time from the estimated travel time (obtained using the Google Maps API (Google, Mountain View, CA, USA) based on time of day and the origin and destination of the trip), we can estimate this person’s exposure to traffic congestion for each trip.

Recall that the primary goal of this research is to compare individual exposures to traffic congestion obtained from two assessments: one that only considers commuting trips and one that also considers individuals’ activity-travel patterns in addition to considering commuting trips. Assume that the person in this example is exposed to traffic congestion for 10 min for each of the five trips. An assessment that only considers the two commuting trips (Trips 1 and 2) estimates that the duration of exposure to traffic congestion is 20 min (10 + 10), while an assessment that also considers the non-commuting trips (Trips 3, 4, and 5) as well as the commuting trips (Trips 1 and 2) estimates that the duration of exposure to traffic congestion is 50 min (10 + 10 + 10 + 10 + 10).

**Figure 3:**
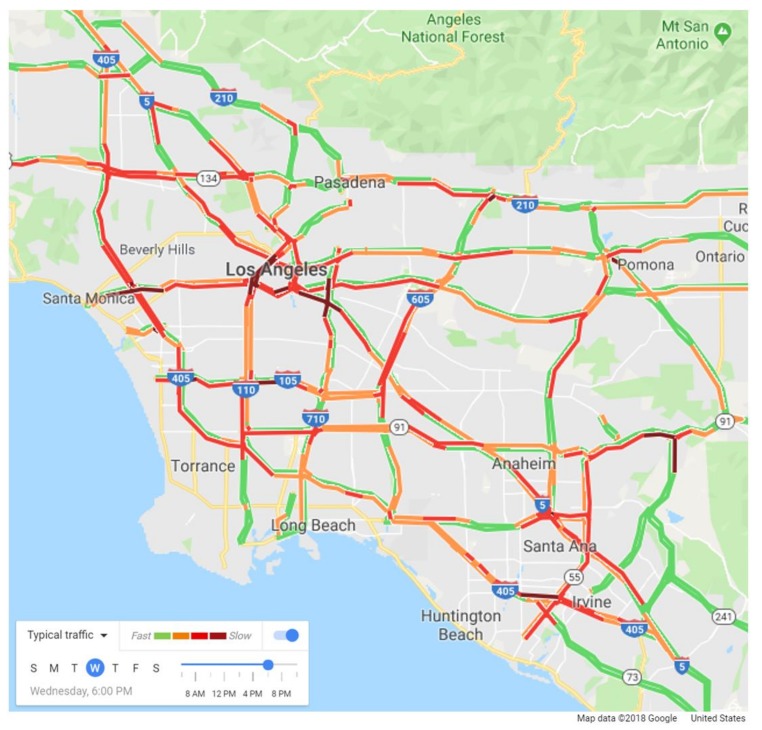
A screenshot of a map of typical traffic congestion levels at 6 P.M. on Wednesday in the Los Angeles Metropolitan Area (Source: Google Maps).

To obtain free-flow travel time and estimated travel time, we utilized the Google Maps API (Figure 3). The Google Maps API estimates driving time between two points when API users provide departure time, departure/arrival/waypoints locations, and route options (e.g., avoiding toll roads or highways) [48,49]. The API computes driving time based on two data sources: (1) crowdsourced real-time traffic data that were submitted by anonymous drivers who consent to send their location information to Google Maps via their smartphones and (2) historical traffic flow databases that Google has established [48]. Free-flow travel time is derived as if trips occurred at 2 A.M. when traffic volumes practically approach 0. To the best of our knowledge, while no study has compared the accuracy of Google Maps data with those from other sources, it seems that travel times provided by Google Maps are highly accurate based on several sources on the web. For instance, in one assessment that used 56 trips with an average journey time of 32 min, the average travel time difference between actual and estimated travel times is 1.8 min (see https://blog.ancoris.com/how-accurate-is-google-maps-journey-time).

In this research, the departure time and geographic coordinates (e.g., longitude and latitude) of the origin and destination of each trip of the participants recorded in the travel survey were used to obtain free-flow travel time and estimated travel time through the Google Maps API. Note that we did not use the reported travel times from the survey as the actual travel time in this study because the survey did not provide the travel routes of participants’ trips and it is not possible to estimate the corresponding free-flow travel time for each of the participants’ trip, which in turn renders the comparison between the free-flow travel time and the (estimated) travel time that considers traffic congestion for each trip of the participants impossible. Further, estimating actual travel time using the Google Maps API serves to avoid the recall and rounding errors common in the reported travel times of travel surveys. 

Using the Google Maps API service has several advantages over traditional desktop-based GIS programs. One compelling advantage is that using the API service does not require researchers to prepare a considerable amount of data and use considerable computing resources. For example, the Google Maps API promptly provides users with travel time that considers traffic congestion between any two given locations (i.e., longitude, latitude) at 20-min intervals.

To obtain this detailed travel time estimate, conventional desktop-based GIS programs require researchers to prepare a considerable amount of network data (e.g., [50,51]). For example, researchers need to prepare road network files, estimate the traffic volume and speed on each road segment at each time interval, and generate penalty information for each street intersection (e.g., one-way roads, no-left-turn penalty, and so on). Preparing these datasets may not be feasible for large metropolitan areas such as Los Angeles. Additionally, even if researchers can prepare the required data, it may take substantial time to run the shortest-path algorithm since the road networks are large and complex. However, by using the Google Maps API service, researchers only need to develop a simple program based on easily accessible programming languages (e.g., Python, Java, and so on). Moreover, since the calculations of travel time are performed inside the API service (where the API uses its own high-performance computing facilities), researchers can get results immediately. For these reasons, there has recently been a growing number of studies that extensively employed the Google Maps API and other map-based API services (e.g., [52,53]).

However, it should be noted that the Google Maps API service has several limitations. One limitation is that users may not know the detailed mechanism of how it estimates travel time. However, documentation from API service providers may mitigate this issue (e.g., [49]). Another limitation is that API services may charge a fee based on the number of API requests. For example, Google Maps API users can use 40,000 API requests per month for free. Beyond the 40,000 free requests, users need to pay a fee per single API request (e.g., a single query of travel time estimation for a single pair of origin and destination) [49]. Thus, the Google Maps API service may not be a viable option for researchers who want to obtain travel times for a larger number of origin-destination pairs [54]. However, this limitation did not significantly affect our research because we did not need a large number of requests; we requested travel time estimates for approximately 1000 trips, which the 250 selected participants undertook. 

## 4. Results

In the first part of Section 4.1 below, we explore whether traffic congestion intensities are different across space and time in the study area based on data from the INRIX 2017 Global Traffic Scorecard [29]. In the second part of Section 4.1, we examine how traffic congestion levels are different over space and time based on the 1022 trips made by the 250 selected participants of the survey. In Section 4.2, we compare two assessments of individual exposures to traffic congestion for the 250 participants: one that considers only commuting trips and the other one that considers both commuting and non-commuting trips. In Section 4.3, we explore how three individuals from the same household are exposed to traffic congestion in unique ways over space and time through visualizations of their space-time trajectories.

### 4.1. Spatiotemporal Variations in Traffic Congestion Intensities

In this section, we answer the first research question: Do spatiotemporal variations exist in traffic congestion intensities? We empirically examine whether traffic congestion intensities are different across space and time in the study area based on data from the INRIX 2017 Global Traffic Scorecard [29] and the trips made by the 250 selected participants. Using the first data source, traffic congestion intensity is assessed in terms of the percentage of congestion travel time that drivers experience out of gross travel time; using the second data source, traffic congestion intensity is derived as the ratio of travel time that considers traffic congestion to free-flow travel time. This is an important question because spatiotemporal variabilities in traffic congestion intensities may exacerbate the UGCoP. Although it is widely known that traffic congestion intensities are different over space and time [55], here we empirically investigate its precise spatiotemporal configurations in the study area.

First, we investigate general spatiotemporal variations of traffic congestion intensities in the study area using data from the INRIX 2017 Global Traffic Scorecard, which provides data on traffic congestion for over 1360 cities around the world (Table 2) [29]. Although the Los Angeles Metropolitan Area is globally notorious for its severe traffic congestion, and as these data indicate, we can observe the spatiotemporal heterogeneity of the traffic congestion in the area: (1) There are temporal variations in traffic congestion. For instance, inter-city drivers experience traffic congestion for 22% of their gross travel time during peak hours while 10% of their gross time occurs during non-peak hours (e.g., around noon) [29]. (2) There are spatial variations in traffic congestion. For example, during non-peak hours, intra-city drivers experience traffic congestion for 13% of their gross travel time, while inter-city drivers experience traffic congestion only for 10% of the gross travel time [29]. 

**Figure 4:**
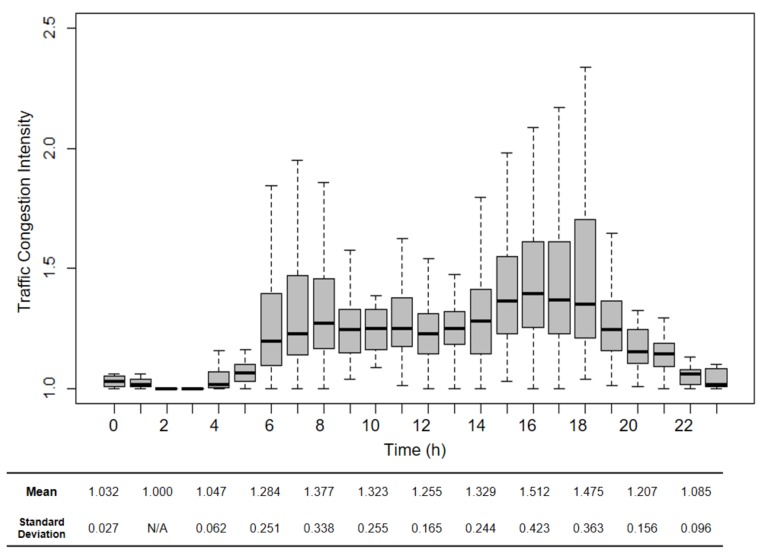
Observed traffic congestion intensity over space and time.

Second, we examine the spatiotemporal variations in traffic congestion intensities based on the 1022 trips made by the 250 selected participants (note that, here, traffic congestion intensity for a trip is derived as the ratio of travel time that considers traffic congestion to free-flow travel time; see Figure 2 and earlier description on how these two travel times for each trip are derived). Figure 4 illustrates traffic congestion intensity (the vertical axis) variations of these 1022 trips by trip departure time (the horizontal axis). The ratio of congestion and free-flow travel times is widely used in practice to represent the severity of traffic congestion at the road-segment level (e.g., [29,46,47]). For example, if traffic congestion intensity is 1.5, it means that the travel time that considers traffic congestion is 1.5 times longer than the free-flow travel time due to traffic congestion. Therefore, the minimum value of the traffic congestion intensity is 1.0 because free-flow travels give the minimum travel time.

We can observe the following two things in Figure 4. First, there are temporal variations in traffic congestion intensity. Not surprisingly, traffic congestion is generally severe during peak hours (e.g., 7–9 A.M., 3–6 P.M.). Moreover, although traffic congestion during non-peak hours is less severe than that of peak-hours, traffic congestion is still observed at any time during a day. This corroborates our earlier observation that there are temporal variations in traffic congestion in the Los Angeles Metropolitan Area. Second, there are spatial variations in traffic congestion intensity. Vertical distributions of the observations (i.e., indicated by the range of the boxes) represent various traffic congestion intensities at different locations at each hour. For example, the range of the box at 6 P.M. is wider than that at 12 P.M., which means more spatial variations in traffic congestion intensities exist at 6 P.M.

These two findings answer our first research question: Do spatiotemporal variations exist in traffic congestion intensities? Based on the observations made from Table 2 and Figure 4, it is clear that spatiotemporal variations of traffic congestion intensities exist in the study area. In what follows, we continue our analysis to compare individuals’ exposures to traffic congestion obtained from two assessments based on the trips made by the 250 selected participants: one that only considers commuting trips and one that considers both commuting and non-commuting trips (i.e., taking into account individuals’ activity-travel patterns).

### 4.2. Differences in Individual Exposures to Traffic Congestion between the Two Assessments

We conduct a paired sample *t*-test to see whether individual exposures to traffic congestion for each participant are significantly different between the commute-only assessment and the assessment that also considers participants’ activity-travel patterns (i.e., considering both commuting and non-commuting trips) (please see Section 3.2.2 and Figure 2 for a detailed explanation of the method). Here, individual exposure to traffic congestion for each trip is estimated by subtracting its free-flow travel time from its estimated travel time (which considers traffic congestion and is estimated using the Google Maps API). This is the additional travel time for a trip due to traffic delay or congestion. 

For each participant, we obtain a commute-only exposure measure by adding the additional travel times incurred by the commuting trips and another exposure measure by adding the additional travel times of both commuting and non-commuting trips. We then compare the difference between these two exposure measures for each participant (and thus a paired sample *t*-test is used). 

**Figure 5:**
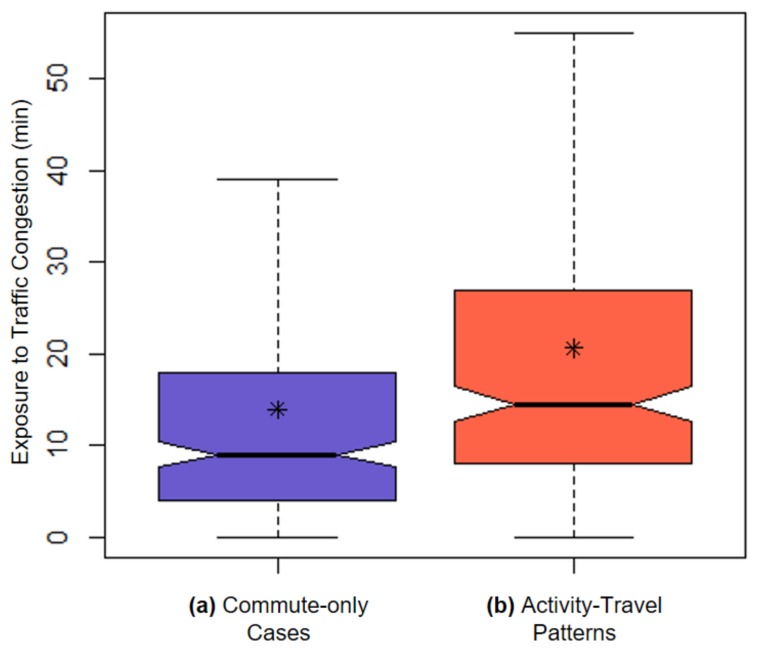
Box plots of individuals’ traffic congestion exposure for (**a**) the commute-only assessment and (**b**) the assessment that also considers participants’ activity-travel patterns. (Outliers are not presented.)

Table 3 indicates that the mean difference in participants’ exposure to traffic congestion is 6.66 min, which means that the duration for which a participant experiences traffic congestion increases on average by 6.66 min (47.78%) when participants’ activity-travel patterns are considered, compared to the commute-only assessment. The result of the paired sample *t*-test confirms that the differences in exposures to traffic congestion between the two assessments are statistically significant (*p* < 0.001). Figure 5 visualizes the results presented in Table 3. The box plots also show that the average exposure to traffic congestion when individuals’ activity-travel patterns are considered is higher than the average exposure obtained in the commute-only assessment.

**Figure 6:**
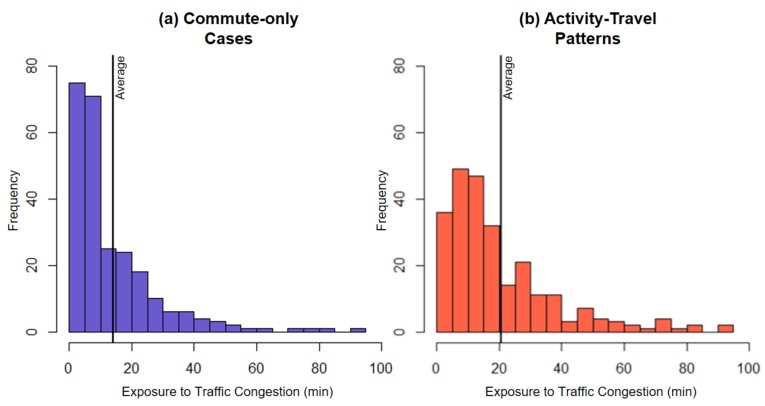
The histograms of traffic congestion exposure in (**a**) the commute-only assessment and (**b**) the assessment that considers participants’ activity-travel patterns.

Moreover, we examine the probability distribution function of individual exposure to traffic congestion. Figure 6 presents the histograms of individuals’ traffic congestion exposure levels for the two assessments. As the histograms show, when individuals’ activity-travel patterns are considered (right histogram), the shape of the probability distribution function becomes less skewed and converges to its mean. Table 4 shows that skewness (from 2.313 to 1.724) and kurtosis (from 9.766 to 5.982) of the histogram decrease after activity-travel patterns are considered. The results indicate that the probability distribution function of individual exposures to traffic congestion shows a tendency to converge to its average when individuals’ activity-travel patterns are considered.

This phenomenon can also be understood as a manifestation of the neighborhood effect averaging problem (NEAP) observed by Kwan [36]. However, there are two important differences between our observations here and the original interpretation of the NEAP put forward by Kwan [36]. First, both exposure assessments (i.e., one that considers only commuting trips and one that considers people’s entire activity-travel patterns) in this research are mobility-based. In other words, the commute-only exposure assessment is not residence-based because it already included some portion of individuals’ daily mobility (i.e., commuting trips). However, the original articulation of the NEAP compares residence-based exposures with mobility-based exposures. This indicates that the NEAP can also be encountered in environmental exposure assessments when only parts (instead of all) of people’s daily mobility are ignored.

Second, the probability distribution functions of the two exposure assessments in this study are not bell-shaped but heavily skewed. The original notion concerning the NEAP only focuses on distributions of individual exposures that approximate a bell-shaped distribution (one such distributions is the normal distribution), but distribution functions in our research are heavily skewed. This indicates that the neighborhood effect averaging problem can also be encountered when the probability distributions of individual exposures are not bell-shaped. These two differences between our observations here and the original interpretation of the NEAP extends the original interpretation of the NEAP in important ways.

**Figure 7:**
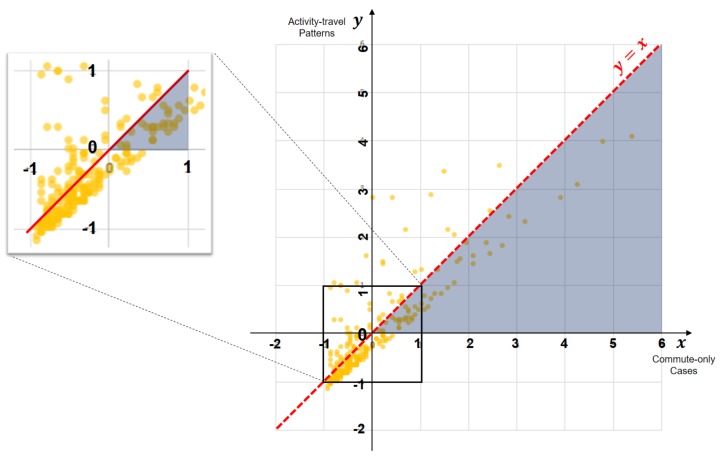
Standardized (*z*-score) individual exposures to traffic congestion.

Additionally, we examine in detail how considering individuals’ activity-travel patterns impact the relative levels of individual exposure to traffic congestion. Figure 7 illustrates the standardized (*z*-score) individual exposures to traffic congestion. The horizontal axis displays individual exposures of the commute-only cases, while the vertical axis represents individual exposures obtained by the assessment that considers participants’ activity-travel patterns. For example, points in the first quadrant (top-right) represent cases when individual exposures to traffic congestion are higher than the average in both assessments. On the contrary, points in the third quadrant (bottom-left) indicates that individual exposures to traffic congestion are lower than its average in both assessments. A closer examination of the graph yields a couple of important findings.

First, the standardized (*z*-score) individual exposures to traffic congestion of most participants (203 participants, 81% of the selected subsample) range between −1 and 0 (see the focused area in the inset). Second, a majority of participants (48 out of 69) in the first quadrant are located in the blue triangular area. This indicates that individual exposures shift much closer to its mean value when activity-travel patterns are considered. These findings confirm an earlier observation that many individuals have exposure levels around the average value while fewer individuals have very high or low exposure levels, and considering individuals’ activity-travel patterns leads the exposure level to converge to its mean [36].

Based on these results, we also answer the second question: Will exposures to traffic congestion based on commute-only assessments be significantly lower than those obtained from assessments that also consider individuals’ activity-travel patterns in addition to commuting trips? We found that this is indeed the case. There are statistically significant differences between exposures evaluated with and without considering individuals’ activity-travel patterns, indicating that the UGCoP is a serious issue. We also found that ignoring individuals’ activity-travel patterns may exacerbate the NEAP. Therefore, we can conclude that overlooking people’s activity-travel patterns may lead to serious methodological issues in the form of the UGCoP and the NEAP when assessing their exposures to traffic congestion. 

### 4.3. Space-Time Visualizations of Individual Exposures to Traffic Congestion

**Figure 8:**
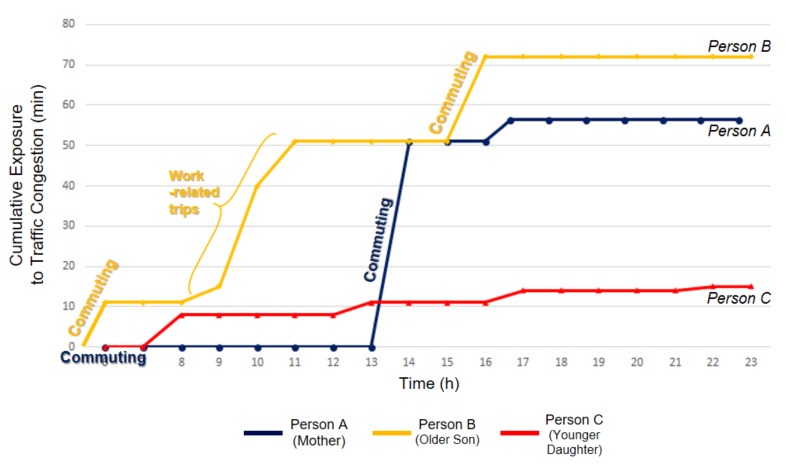
Cumulative exposures to traffic congestion of three selected individuals from the same household (A: Mother, B: Older Son, C: Younger Daughter).

**Figure 9:**
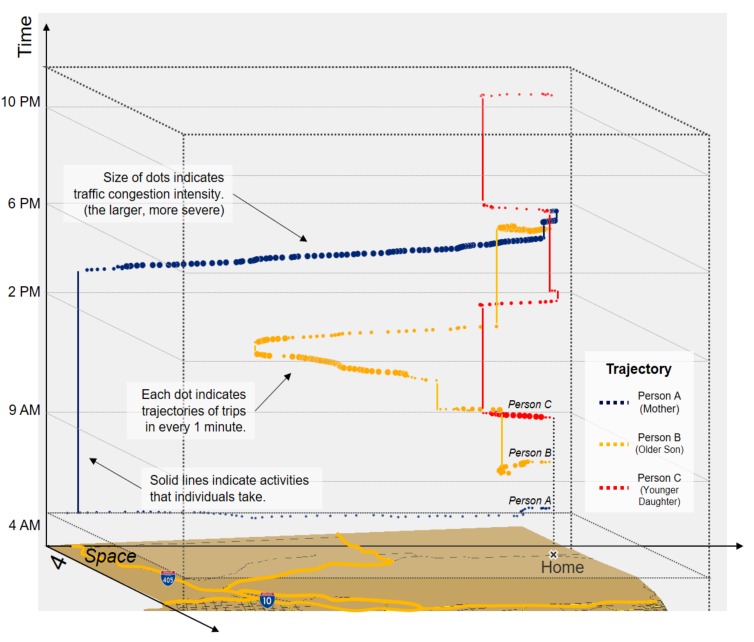
Space-time visualizations of traffic congestion exposures of three individuals from the same household (A: Mother, B: Older Son, C: Younger Daughter).

Figure 8 illustrates the cumulative traffic congestion exposures of 3 individuals from the same residence (i.e., the same household) over the 24 hours of the survey day. Line A (blue) indicates the mother’s exposure to traffic congestion, while Lines B (yellow) and C (red) represent the older son and the younger daughter respectively. Also, space-time visualizations of these individuals’ activity-travel patterns are presented in Figure 9. The vertical axis (*t*) represents time, and the horizontal plane displays space (*x*, *y*). Each dot represents a 1-min interval in the trip trajectories obtained from the Google Maps API. The size of the dots indicates traffic congestion intensity. For instance, larger dots represent more intense traffic congestion. The vertical solid lines indicate durations when individuals are performing activities at fixed locations, as their location (*x*, *y*) does not change over time.

The space-time illustration of individuals’ trajectories clearly shows that individuals are exposed to traffic congestion in unique ways over space and time. First, although all 3 individuals have a similar travel-demand environment (e.g., actively employed and driving their own cars), each family member’s traffic congestion exposure varies because of their idiosyncratic activity-travel patterns.

As Figure 9 shows, for example, the traffic congestion exposure of Person A (mother) ranges between 10 and 70, while that of Person C (younger daughter) varies between 10 and 20. This difference can be explained by the different activity-travel patterns between these two persons. Person A takes longer commuting trips, and she is heavily exposed to traffic congestion especially during her way back home. Person A is exposed to severe traffic congestion when she takes non-commuting trips, but the non-commuting trips do not significantly contribute to the total exposure because the length of the trips is relatively short. By contrast, Person C is less exposed to traffic congestion than Person A. Most trips that Person C takes are near her residence and relatively short, which enables her to avoid heavy exposure to traffic congestion.

Specifically, we can observe that the younger son (Person B) is exposed to heavier traffic congestion than the others in the household. His traffic congestion exposure becomes more severe when his activity-travel patterns are considered. This drastic increase is mainly because of his work-related trips happening near areas in South Los Angeles during non-peak hours (e.g., 9 A.M.–2 P.M.), when traffic congestion there is still severe.

**Figure 10:**
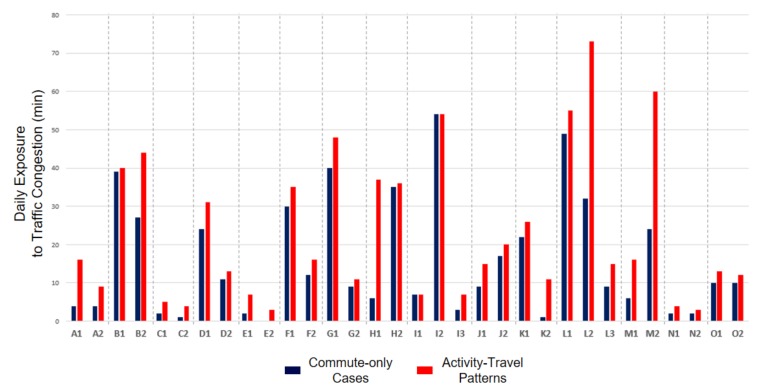
Individual exposures to traffic congestion of 32 individuals from 15 households in the subsample (Same alphabet means the same household.)

Further, Figure 10 depicts the exposures to traffic congestion of 32 individuals from 15 households in the subsample for both the commute-only assessments and assessments that also consider their activity-travel patterns. The bar graph clearly shows that individuals from the same household are differently exposed to traffic congestion because individuals’ activity-travel patterns are idiosyncratic.

Based on these results, we answer the third question: How are individuals uniquely exposed to traffic congestion as a result of their activity-travel patterns? The results confirm that individuals are idiosyncratically exposed to traffic congestion due to their distinctive activity-travel patterns. The results also corroborate previous studies [37,56], which argue that individuals from the same household are differently exposed to environmental influences or contexts. Therefore, these results may cast doubt on the validity of previous studies’ exclusive focus on commuting trips during peak hours and using data aggregated over areas (e.g., census tracts) because they did not fully reflect individuals’ unique activity-travel patterns.

## 5. Conclusions

This research empirically examined whether the uncertain geographic context problem (UGCoP) and the neighborhood effect averaging problem (NEAP) are encountered in research on individual exposures to traffic congestion. We used crowdsourced real-time traffic congestion data and activity-travel data of 250 individuals in Los Angeles to compare two assessments of individual exposure to traffic congestions: one that only considers commuting trips and one that also considers individuals’ non-commuting trips in addition to considering commuting trips (thus taking people’s activity-travel patterns into account).

First, the results indicated that spatiotemporal variations in traffic congestion intensity exist in the study area, which calls for the consideration of individuals’ activity-travel patterns when assessing their exposures to traffic congestion in future research. 

Second, the paired sample *t*-test results revealed that individual exposures to traffic congestion are significantly underestimated when individuals’ activity-travel patterns are ignored. Further, the results highlighted that the probability distribution function of individual exposures is heavily skewed but tends to converge to its average value when individuals’ activity-travel patterns are considered, which is a manifestation of the neighborhood effect averaging problem (NEAP). These results indicated that both the UGCoP and the NEAP are critical methodological issues in traffic congestion and health studies.

Lastly, we presented space-time visualizations of the traffic congestion exposures of 3 individuals from the same household. The results illustrated that since individuals have idiosyncratic activity-travel patterns, their exposures to traffic congestion vary significantly even if they live at the same residential location. These results corroborate the claim in previous studies that using residence-based methods or data aggregated over areas (e.g., census tracts) may lead to erroneous assessments of individual exposures to traffic congestion or other environmental influences [31].

The results of our research imply that epidemiological studies should pay more attention to individuals’ activity-travel patterns when assessing people’s environmental exposures. As the results of this study illustrated, ignoring individuals’ daily mobility (i.e., activity-travel patterns) may result in erroneous assessments of their exposures to traffic congestion. Eventually, using inaccurately estimated traffic congestion exposures may exacerbate inferential errors or lead to significantly misleading results when investigating the effects of traffic congestion exposures on human health. In addition, the results imply that researchers who study environmental exposures should focus more on individual-level analysis, since this study shows that people who live in the same residential location may have different traffic congestion exposures due to their distinct activity-travel patterns. This implies that using residence-based methods or data aggregated over areas (e.g., census tracts), which are popular approaches in previous studies, may lead to critical methodological issues.

**Figure 11:**
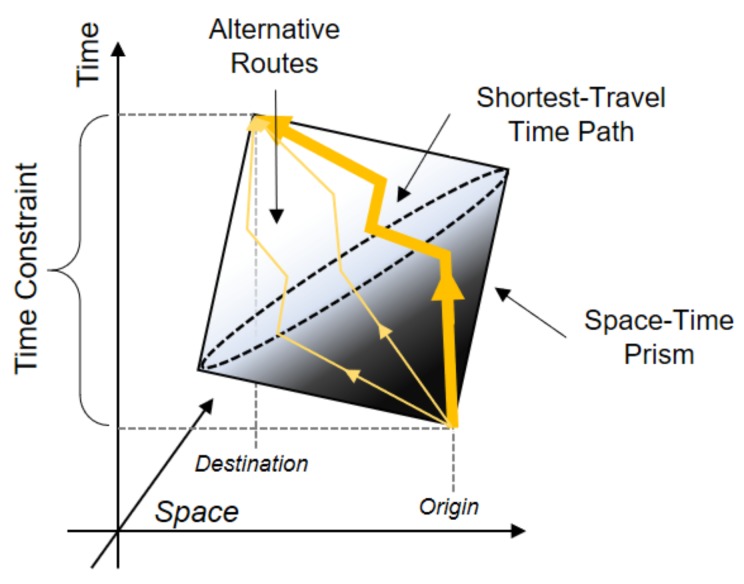
An example of a space-time prism.

Although this study significantly advances our knowledge about two critical methodological issues (i.e., the UGCoP and the NEAP) in traffic congestion and health studies, it has several limitations that should be addressed in further studies. First, we presumed that individuals used the shortest path (in terms of travel time) to travel from one location to another, which may not fully capture their true activity-travel patterns. Although this assumption is reasonable, people may not necessarily use the shortest path. One possible solution to this issue may be to employ a space-time prism [57], as illustrated in Figure 11. A space-time prism consists of points that individuals may possibly visit given their spatiotemporal constraints [50,58]. Considering possible alternative routes in a space-time prism may help researchers comprehensively assess traffic congestion exposures. 

Second, further studies need to consider the subjective aspects of individual exposures to traffic congestion. Previous studies revealed that exposures to traffic congestion may go through subjective perception filters [12,16,59]. This indicates that although people are exposed to the same level of objective traffic congestion (e.g., 20 min in traffic congestion), the effects of the objective traffic congestion on health may follow different mechanisms for each individual. However, due to the limitations of the survey data used in this study (which did not collect or provide any data on participants’ perceptions), we were not able to address this issue. One possible solution may be to integrate in-depth interviews about subjective factors with activity-travel surveys [25,60]. By combining the subjective experiences of traffic congestion with objective measures, future research may further advance our knowledge of the health impacts of exposures to traffic congestion. 

Third, more scrutiny is required to unveil the temporal dimension of the effects of traffic congestion exposures on health [24]. We computed total traffic congestion exposure in minutes because we presumed that cumulative exposure may influence health. However, the effects of traffic congestion exposures on health may show “time-lagged response” [24], which means that it may have health effects afterward. Moreover, not only the duration of traffic congestion but also the variability in driving time may negatively affect health. Since several epidemiological studies reported such evidence [15,61], more attention is required to clearly understand the temporal aspect of the effects of traffic congestion on health. One possible solution is to utilize real-time global positioning system (GPS) technology to gain clearer pictures and more detailed understanding of these temporal effects of traffic congestion on health [62,63].

## Figures and Tables

**Table 1:** Comparison of the sociodemographic attributes of the 250 selected participants with those of the larger population of the Los Angeles Metropolitan Area.

Sociodemographic Attributes	The 250 Selected Individuals				Los Angeles Metropolitan Area ^(a)^			
Gender	Male		Female		Male		Female	
	53%		47%		55%		45%	
Race	White	African-American		Asian	White	African-American		Asian
	59%	6%		14%	58%	6%		17%
Age (mean)	45.2 years old				39.9 years old			
% of people with higher education ^(b)^	62%				65%			

^(a)^ American Community Survey (ACS) 2016 5-year estimates, ^(b)^ Higher education indicates education attainment that is equal to or higher than bachelor’s degree.

**Table 2:** Spatiotemporal variations in traffic congestion in the study area (Source: [29]).

Types of Trips	Peak Hours	Non-Peak Hours (Daytime)	Non-Peak Hours (Late Nighttime)
Intra-city trips ^(b)^	20% ^(a)^	13%	8%
Inter-city trips ^(c)^	22%	10%	3%

^(a)^ Percentage of congestion travel time that drivers experience out of gross travel time. ^(b)^ Intra-trips refer trips that occur within the city. ^(c)^ Inter-trips refer trips that occur into/out of the city.

**Table 3:** Mean exposure to traffic congestion in the commute-only assessment and the assessment that also considers participants’ activity-travel patterns.

Statistics	Commute-Only Assessment	Activity-Travel Patterns Considered	Mean of Differences ^(a)^
Mean	13.94 (min)	20.60 (min)	6.66 *** ^(b)^
Standard Deviation	14.87	18.13	-

^(a)^ Paired sample *t*-test result, ^(b)^ *** *p*-value < 0.001.

**Table 4:** Skewness and kurtosis of the histograms for both assessments.

Statistics	Commute-Only Assessment	Activity-Travel Patterns Considered
Skewness	2.313	1.724
Kurtosis	9.766	5.982
